# Melanoma characteristics in Brazil: demographics, treatment, and survival analysis

**DOI:** 10.1186/s13104-015-0972-8

**Published:** 2015-01-16

**Authors:** Vinicius de Lima Vazquez, Thiago Buosi Silva, Marcelo de Andrade Vieira, Antônio Talvane Torres de Oliveira, Marcílio Vital Lisboa, Diocésio Alves Pinto de Andrade, José Humberto Tavares Guerreiro Fregnani, Estela Cristina Carneseca

**Affiliations:** Department of Surgery, Melanoma/Sarcoma, Barretos Cancer Hospital, Rua Antenor Duarte Vilela, 1331, 14784-400 Barretos, São Paulo Brazil; Research and Teaching Institute, Barretos Cancer Hospital, Barretos, São Paulo Brazil; CPOM, Molecular Oncology Research Centre, Barretos Cancer Hospital, Barretos, São Paulo Brazil; Barretos School of Health Sciences, Dr. Paulo Prata-FACISB, Barretos, São Paulo Brazil; Oncology Unit, Marcio Cunha Hospital, Ipatinga, Minas Gerais Brazil; InORP, Oncologic Institute, Rua Ayrton Roxo, 571 CEP, 14025-270 Ribeirão Preto, São Paulo Brazil; Proestat Statistical Institute, Ribeirão Preto, São Paulo Brazil

**Keywords:** Brazil, Melanoma, Cutaneous malignant, Latin America, Prognosis, Sentinel lymph node biopsy, Skin neoplasms

## Abstract

**Background:**

The incidence of melanoma, one of the most aggressive of the skin cancers, has been increasing worldwide in the last few decades. Data from Latin America and Brazil remain scarce. We aimed to describe the demographic, clinical, and histopathological data; therapy characteristics; and survival rates of the Brazilian melanoma patient population.

**Results:**

We collected and analysed retrospective data from 15 years at a tertiary cancer centre. We describe patient characteristics and treatment. We calculated survival, and identified the main prognostic factors through univariate and multivariate analysis. We analysed a total of 1073 patients, with a mean age of 56.7 years. Men and women experienced similar prevalence, and 91.2% of patients had white skin. The most prevalent subtype was superficial spreading, and the most prevalent anatomic location was the trunk (32.2%), followed by the lower extremities (28%). Of all cases, 567 (52.9%) were assigned to clinical stages I and II, while 382 (32.6%) were stages III and IV. Surgery was the main treatment. Sentinel node biopsy was performed in 373 patients, with 23.8% positivity. Overall actuarial 5-year survival was 67.6%. Multivariate analysis showed that gender, serum lactate dehydrogenase (LDH) levels at diagnosis; anatomic location, TNM stage, and local recurrence were significant prognostic factors.

**Conclusions:**

Overall survival was lower than worldwide rates. The main factors influencing survival were similar to those in other populations. Local recurrence was independently associated with lower survival rates. The high prevalence of advanced cases reinforces the importance of strategies to diagnose melanomas in the early stages. There is a need for future multi-institutional prospective studies to attain a better understanding of possible socioeconomic and other influences on survival among melanoma populations in Brazil and Latin America.

## Background

Melanoma is a neoplasm arising from melanocytes [1] in the skin, mucosa, or uvea. Melanoma constitutes less than 5% of skin cancers but is responsible for around 95% of skin cancer deaths, and its incidence has been rising worldwide over recent decades [2]. In the United States, approximately 70,000 cases are diagnosed every year, with approximately 9000 deaths. In Brazil, it is estimated that 6000 new cases occur each year, resulting in 1300 deaths [3-5]. Risk factors for melanoma development include exposure to ultraviolet (UV) rays, as well as individual phenotypical characteristics, such as fair skin/hair pigmentation, presence of multiple nevi, immunosuppression, and family history [6].

Melanoma prognosis is based on clinical and histopathological factors. For localized disease, the primary tumour thickness (Breslow) is the most important prognostic factor. Tumour ulceration, mitotic rate, gender, age, and serum levels of lactate dehydrogenase (LDH) are also related to prognosis [7-10]. For metastatic disease, the presence and characteristics of lymph nodes and distant metastases are the major factors impacting survival. Lymph node tumour burden and the diagnosis of micrometastases by sentinel node biopsy (SNB) have been shown to be closely associated with prognosis. Although SNB is not intended as therapy, its results often change therapeutic plans. In standard practice, patients with positive SNBs, as well as patients with clinically diagnosed stage III disease, undergo lymph node dissection [11-14].

The different characteristics of melanoma and their relation to prognosis have been extensively reviewed [7,8,10,15]. In 2001, melanoma became the first cancer to be staged using an evidence-based system created on an extensive multi-institutional database (more than 17,000 melanoma cases) by the American Joint Committee on Cancer and the International Union Against Cancer (UICC) [8]. Unfortunately, this database included no Latin American or Brazilian patients. Skin phenotype and sun exposure are factors associated with melanoma incidence, while early diagnosis and easy access to healthcare are related to the prognosis. The Brazilian population is made up of multiple races, with many people of more than one racial heritage, and, hence, a range of skin colours. The population is frequently exposed to UV radiation. Access to healthcare is often difficult, especially for those living in rural areas. The Latin America and/or Brazilian published data are limited to descriptive studies or related to treatment in small series, and none has investigated survival or prognosis [16].

We sought to characterize Brazilian melanoma patients according to demographic, clinical, and histological data, as well as treatment, and to analyse the main factors related to prognosis.

## Results

The study included 1073 patients, whose demographic characteristics are presented in Table 1. The main clinical and histopathological features are shown in Table 2 and treatment characteristics in Table 3. Sao Paulo State was the origin of 781 (72.8%) patients, followed by Minas Gerais with 128 (11.9%). The more distant areas—the North, Northeast, and Central Brazil area—accounted 151 cases (15%). From Sao Paulo State, 160 patients were from the Barretos area (20.5%) and 621 (79.5%) from other areas. The anatomical and histological subtype distributions of primary tumours differed according to skin colour characteristics. Compared with patients with white skin, a higher proportion of those with non-white skin presented with melanomas in the lower extremities (27.9 versus 54.1% p < 0.001) and more of the acral subtype (5.6 versus 18.5% p = 0.002). Of 34 non-white patients with tumours in the lower extremities, 10 presented with acral lentiginous melanomas (29.4%), 11 with nodular (32.4%), seven with superficial spreading (20.6%), one with lentigo maligna (2.9%), and five other/not otherwise specified (14.7). Women presented with more melanomas in the lower extremities than men (36.3 versus 23.2%; p < 0.001), and fewer in the trunk (29 versus 40%; p < 0.001). The superficial spreading histologic subtype was generally more prevalent among women (43.6 versus 33.7%; p = 0.035), while the nodular subtype was more common among men (34.6 versus 28.5%; p = 0.035).

**Table 1:** **Demographics of 1073 melanoma patients**

**Patient characteristics**	
**Gender,** ***n*** **(%)**	
Male	543 (50.6)
Female	530 (49.4)
**Skin colour,** ***n*** **(%)**	
White	978 (91.2)
Other	87 (8.1)
Not available	8 (0.7)
**Age, range (mean, SD)**	1-95 (56.7, 16.0)

**Table 2:** **Main clinical and histological characteristics of 1073 melanoma patients**

**Characteristics**	**Number**	**%**
**Anatomical location (primary tumour)**		
Trunk	347	32.2
Lower extremities	300	28.0
Head and neck	197	18.4
Upper extremities	149	13.9
Mucosa	3	0.3
Multiple	2	0.2
Unknown	9	0.8
Not available	66	6.2
**Histological subtype**		
Superficial spreading	353	32.9
Nodular	288	26.8
Acral lentiginous	76	7.1
Lentigo maligna melanoma	40	3.7
Other/not classified	156	14.6
Not available	160	14.9
**Tumour depth (Breslow)**		
Up to 1.0 mm	225	21.0
1.1 to 2.0 mm	184	17.1
2.1 to 4.0 mm	171	15.9
More than 4.0 mm	231	21.5
Not available	262	24.4
**Clark classification**		
II	110	10.3
III	269	25.1
IV	350	32.6
V	129	12.0
Not available	216	20.0
**Ulceration**		
Present	307	28.6
Absent	371	34.6
Not available	395	36.8
**Mitotic index**		
Up to 1 mitosis/mm^2^	104	9.7
More than 1 mitosis/mm^2^	260	24.2
Not available	709	66.1
**Lymph node status**		
N0	615	57.3
N1	87	8.1
N2	72	6.6
N3	87	8.1
Not available	212	19.8
**Clinical stage (AJCC)**		
I	296	27.6
II	271	25.3
III	214	19.9
IV	168	15.7
Not available	124	11.6

**Table 3:** **Treatment characteristics of 1073 melanoma patients**

**Treatment**	***n***	**%**
**Surgery (primary tumour)**		
Not operated	135	12.6
Primary closure	385	35.8
Local surgery*	257	20
Local surgery and reconstruction (graft or flap)	213	23.8
Amputation	64	6.0
Second intention healing	19	1.8
**Sentinel node biopsy**		
No	700	73.8
Yes	373	26.2
Positive	89	23.9
Negative	251	76.3
Not accessed/available	33	8.8
**Adjuvant therapy**		
No	908	84.6
Radiation	37	3.4
Systemic	86	8.0
Not Available	42	3.9
**Systemic therapy**		
No	471	43.9
Yes	180	16.7
Not available	422	39.3
**Radiation therapy (metastasis)**		
No	518	48.3
Yes	133	12.4
Not available	422	39.3

*Reconstructive surgery information not available.

In accordance with international treatment guidelines, surgery was the most common treatment modality, with a few patients receiving adjuvant radiation or systemic therapy. Amputation was mainly used to treat tumours in fingers/toes and advanced tumours of the extremities; no amputations were necessary to treat complications. Of the 135 patients not operated on for the primary tumour, 95 had received previous treatment at other institutions and 60 cases were stage IV; 33 were stage III or featured nodal recurrence. Twenty-nine patients underwent surgery at our institution. Twenty-seven patients received no treatment. These were advanced cases and/or refused treatment. In 373 cases, sentinel node biopsy (SNB) was performed using preoperative dynamic lymphoscintigraphy with 99mTc-labelled sulphur colloid and intraoperative blue dye and a gamma probe [17]. The rate of positivity was 23.8% (89 patients). Thirty-three cases (8.8%) had technical problems, or data were unavailable from the referring site. Among the 74 positive sentinel node cases with known tumour thickness (pT), only four cases (6.3%) were ≤ 1 mm (pT1), 16 (17.2%) were pT2, 18 (23.4%) were pT3, and 36 (41.9%) were pT4 (Table 4). Of the 284 negative cases (76.2%), locoregional recurrence occurred in 28 (9.8%).

**Table 4:** **Tumour thickness in patients submitted to sentinel node biopsy**

**Tumour thickness (pT)**	**Total**	**Positive**	**Negative**
pT1, *n* (%)	63 (16.9)	4 (6.4)	59 (93.6)
pT2, *n* (%)	93 (24.9)	16 (17.2)	77 (82.8)
pT3, *n* (%)	77 (20.6)	18 (23.4)	59 (76.6)
pT4, *n* (%)	86 (23.0)	36 (41.9)	50 (58.1)
Not available, *n* (%)	54 (14.5)	15 (27.8)	39 (72.2)
Thickness in mm, mean (SD)	3.3 (3.2)	5.0 (4.4)	2.7 (2.4)

**Figure 1:**
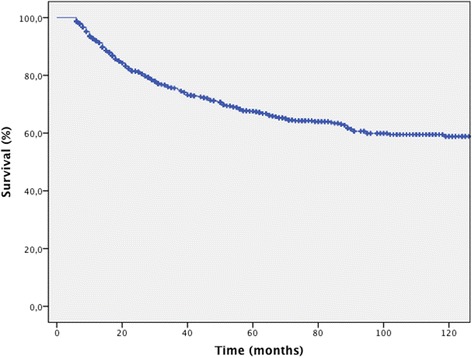
**Cancer-specific survival for all melanoma patients.**

Survival analysis included 915 patients. At the last evaluation, 513 (56.1%) were alive and disease-free, 46 (5.0%) were alive with cancer, 266 (29.1%) had died due to melanoma, 79 (8.6%) had died from other causes, six (0.7%) died due to unknown causes, and five (0.5%) were lost to follow-up. Follow-up duration ranged from 6 to 233 months (mean 52 [SD 45] median, 38 months). For all patients alive at the last evaluation (559 cases), the mean follow-up was 63.5 months (SD 45), median 62 months. In 61.2% follow-up lasted longer than 3 years. Only five patients were lost to follow-up (0.5%). Among all patients, the 5-year disease-specific survival (DSS) rate was 67.6% (Figure 1).

Univariate analysis showed that the following demographic, clinical, and histological variables were related to prognosis: age (p = 0.005; Hazard Ratio = 1.011), gender, location of primary tumour, sentinel node positivity, Clark level, histologic subtype, presence of mitosis, ulceration, and microscopic satellitosis. Vascular and/or lymphatic infiltration were not statistically significant predictors of survival (Tables 5 and 6).

**Table 5:** **Disease-specific survival (DSS) according to demographic categories**

**Variable**	**Categories**	***n***	**5-year DSS (%)**	**p**
Gender	Male	448	59.1	<0.001
	Female	467	75.6	
Skin colour	White	840	67.8	0.784
	Other	68	69.3

**Table 6:** **Disease-specific survival (DSS) according to clinical and histological characteristics**

**Variable**	**Category**	***n***	**5-year SCS (%)**	**p**
Anatomical location	Upper limbs	139	75.9	<0.001
	Trunk	303	72.0	
	Head and neck	172	65.3	
	Lower limbs	264	62.1	
	Unknown	7	50.0	
Sentinel node	Negative	258	80.6	<0.001
	Positive	77	54.9	
Clark	II	97	93.4	<0.001
	III	246	80.2	
	IV	313	67.7	
	V	106	38.3	
Histological subtype	Acral lentiginous	53	50.5	<0.001
	Nodular	258	61.3	
	Superficial Spreading	326	82.3	
	Lentigo maligna	34	87.5	
	Not classified/other	136	86.4	
Mitosis/mm^2^	0-1	95	77.5	0.005
	>1	236	62.2	
Intratumoural lymphocyte infiltration	Yes	227	63.8	0.224
	No	228	71.3	
Peritumoural lymphocyte infiltration	Yes	366	71.3	0.136
	No	162	57.4	
Vascular and lymphatic infiltration	Yes	29	50.0	0.066
	No	321	68.5	
Perineural invasion	Yes	14	68.8	0.974
	No	303	67.9	
Ulceration	Present	260	56.8	<0.001
	Absent	332	78.0	
Regression	Yes	57	73.3	0.863
	No	413	69.2	
Microscopic satellitosis	Yes	44	37.2	<0.001
	No	403	74.0	

According to clinical stage and recurrence status, all TNM variables, presence of locoregional or distant recurrence, and serum LDH levels were associated with prognosis (Table 7). Women had higher survival rates than men (Table 5). TNM stage distribution also differed according to sex, with a higher percentage of women with lower stage disease than men (Table 8).

**Table 7:** **Disease-specific survival (DSS) according to clinical stage and recurrence status**

**Variable**	**Category**	***n***	**5-year DSS (%)**	**p**
T	T1	203	93.1	<0.001
	T2	175	75.9	
	T3	162	59.6	
	T4	204	53.1	
N	N0	577	80.3	< 0.001
	N1	81	52.3	
	N2	63	43.3	
	N3	78	27.8	
M	M0	732	72.4	< 0.001
	M1	105	26.1	
Clinical stage (TNM)	I	277	91.4	
	II	257	70.1	
	III	198	46.5	
	IV	105	26.1	
Distant recurrence	Absent	653	87.9	< 0.001
	Visceral	177	18.4	
	Non-visceral	53	35.9	
Locoregional recurrence	Yes	122	33.5	< 0.001
	No	753	74.8	
Serum lactate dehydrogenase	≤480 IU/L	614	74.0	<0.001
	>480 IU/L	102	55.4

**Table 8:** **Gender distribution according to TNM stage**

**Stage (TNM)**	**Male** ***n*** **(%)**	**Female** ***n*** **(%)**
0 (In Situ)	40 (7.6)	58 (11.1)
I	122 (23.3)	175 (33.3)
II	131 (25.0)	140 (26.7)
III	122 (23.3)	92 (17.6)
IV	109 (20.8)	59 (11.3)

p < 0.001.

**Figure 2:**
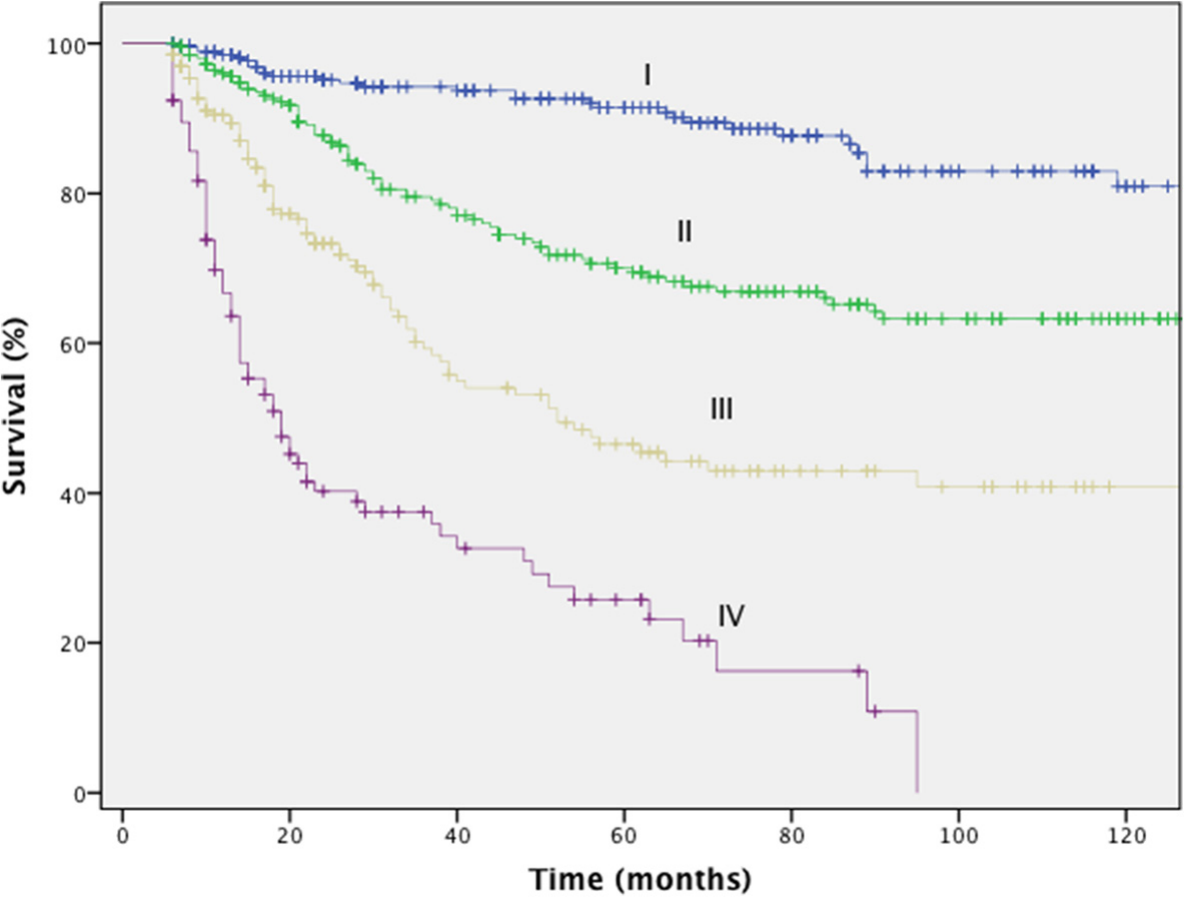
**Melanoma-specific survival according to clinical stage.**

Multivariate analysis demonstrated that gender, TNM stage, LDH levels at diagnosis, the anatomic location of the primary tumour, and locoregional recurrence were associated with prognosis (Table 9) (Figure 2).

**Table 9:** **Multivariate analysis of melanoma-specific survival**

**Variable**	**Category**	***n***	**HR**	**95% CI (HR)**	**p value**
Gender	Male	188	1.000	**-**	**-**
	Female	221	0.508	0.314; 0.822	0.006
Age	**-**	409	1.005	0.988; 1.021	0.582
DHL	**-**	409	1.003	1.001; 1.004	<0.001
Anatomical location	Head and neck	75	1.000	-	-
	Lower limbs	126	0.575	0.305; 1.084	0.087
	Upper limbs	61	0.398	0.181; 0.878	0.022
	Trunk	147	0.429	0.226; 0.814	0.010
Histological subtype	Acral lentiginous	30	1.000	**-**	**-**
	Nodular	149	0.777	0.368; 1.642	0.509
	Superficial spreading	190	0.889	0.399; 1.981	0.774
	Lentigo maligna	14	0.516	0.098; 2.702	0.433
	Not classified/other	26	0.305	0.077; 1.207	0.091
Ulceration	Present	167	1.000	**-**	**-**
	Absent	242	0.902	0.554; 1.468	0.678
T	T1	105	1.000	**-**	**-**
	T2	104	3.005	1.135; 7.957	0.027
	T3	84	2.502	0.862; 7.264	0.092
	T4	116	4.264	1.479; 12.293	0.007
N	N0	310	1.000	**-**	**-**
	N1	34	2.143	1.058; 4.340	0.034
	N2	31	2.163	1.072; 4.367	0.031
	N3	34	3.249	1.772; 5.954	< 0.001
M	M0	395	1.000	**-**	**-**
	M1	14	3.658	1.633; 8.192	0.002
Locoregional recurrence	No	367	1.000	**-**	**-**
	Yes	42	2.198	1.307; 3.696	0.003

## Discussion

For many years, numerous studies have investigated characteristics of melanoma patients and prognostic factors. However, data from Latin America and Brazil remain scarce. The current demographic distribution of melanoma in the Brazilian population is similar to worldwide data [18], with an equal gender distribution, and a mean age of diagnosis at around the sixth decade of life. The majority of patients had white skin (91.4%). However, people in Brazil with moderately brown skin colour typically declare themselves as ‘white’, and our study did not discriminate the skin phototype [19]. Supporting the accurate self-reporting of skin colour, the acral subtype was low among the white-skinned patients (5.5%), which would likely not have been the case with mixed phototypes. The accuracy of self-reporting is also supported by the approximately one-third of non-white-skinned patients presenting with acral lentiginous melanomas.

This study included seven patients under 18 years old; two of whom died from melanoma. Although these patients may have had different predisposing conditions, they were kept in the cohort and in the survival analysis to illustrate the spectrum of the disease.

In terms of patient origin, two-thirds of patients came from Sao Paulo State and of these, only 21% lived in the region of our cancer centre. Considering that Sao Paulo State has 645 cities with total area of 248,222 km^2^, the majority of patients came far from their homes for treatment.

As previously found in Caucasian populations, we observed a prevalence of the superficial spreading histologic subtype, followed by nodular melanoma. The anatomic distribution of lesions showed that the trunk was a more prevalent site in men, and the lower extremities were more prevalent in women, similar to the findings of other epidemiologic studies [18].

Univariate and multivariate analyses showed higher survival rates for women. Interestingly, women more commonly presented in less advanced clinical stages, which was a factor strongly related to higher survival rates.

The used treatment modalities were in accordance with international trends. For localized melanoma, most patients underwent only surgery. The 135 (12.6%) of patients not operated on for the primary tumour are explained mainly by the 14.3% of patients presenting in clinical stage IV and patients presenting with recurrences having had previous treatment at other institutions. Sentinel node biopsy (SNB) was indicated in one-third of patients.

The overall rate of SNB was relatively low, as this procedure was only routinely performed starting in 2003. The number of pT3 and pT4 cases undergoing SNB and the mean thickness (3.2 mm) explains the high (23.4%) positivity rate. pT1 and pT2 positivity was 6.3 and 17.2%, respectively, contrasting with the 23.4% of T3 and 41.9% of T4 tumours, showing the correlation of positivity with thickness. In the literature, the positivity rate varies from 12 to 26% [20-23]. The 9.8% rate of locoregional recurrence among cases with negative SNB is high. However, this rate includes not only nodal lesions but also local recurrence and in-transit metastases. There were a significant number of patients (262, 24.4%), with no Breslow information. Of these, 145 (54%) had stage III and IV disease. SNB was performed in this situation in 54 cases (14.5%). Unfortunately, our centre receives many patients with previous excisional biopsy and no Breslow thickness or other histopathological information. We tried to reanalyse the slides and paraffin blocks, but this was not possible for some cases. In such situations we lowered the threshold for performing SNB for localized disease (clinical stage I and II).

Locoregional recurrence is not usually emphasized, but was strongly related to prognosis, with significantly lower survival rates, which indirectly shows the importance of correct surgical treatment.

Systemic therapy at our institution was chemotherapy and interferon alpha, which was indicated for systemic disease (Stage IV or recurrence) or inoperable Stage III. Surgery for metastatic disease was indicated frequently, mainly for non-visceral disease. First-line chemotherapy was dacarbazine. Second-line was carboplatin and paclitaxel or immunotherapy with interferon alpha. Target therapy or modern immunotherapies are currently in use at the institution, but were not applied to this population.

Radiation therapy was indicated mostly to palliate metastases, and in selected cases, as an adjuvant setting after lymphadenectomy. Indications were bulky tumours (general) and head and neck lymph node metastasis. This course of treatment is not well established but there is evidence for its use in selected cases [24].

The overall 5-year survival rate of 67.6% for all patients was low, but when stratified by clinical stage, it was very similar to other larger series, including the AJCC. These findings show that the higher number of advanced stage cases impacted the overall survival. This is somewhat expected in data from tertiary cancer centres, but is the opposite of findings from developed countries with successful secondary skin cancer prevention policies—related to early diagnosis—which have achieved an overall survival of over 90% [10,25,26].

The presence of intratumoural lymphocyte infiltration, a known factor related to prognosis, did not affect survival rates in this study. Vascular and/or lymphatic infiltration were related to a lower survival rate, but not to a statistically significant degree. Other factors, including mitotic index, sentinel node status, and microscopic satellitosis, associated with prognosis in the univariate analysis, could not be tested in the multivariate model, because a relatively small number of patients remained in the model. The lack of information from referring sites impaired more inclusive analysis. A large number of patients had also had their tumours excised in other centres with little or no histological information on the primary tumour. However, most cases were suitable for univariate analysis and at least 400 cases were pooled in the multivariate analysis.

The overall survival for the entire cohort was lower than that observed in Caucasian melanoma populations. As expected, our data confirmed that the major factors related to melanoma prognosis are TNM stage, LDH levels, and locoregional recurrence. Patients with head and neck tumours had lower survival rates than patients with trunk and upper extremity disease. Anatomic location, besides not being completely accepted as a prognostic factor, has been associated with prognosis in other studies, and was independently related to survival rate in our cohort [15]. Other well-known prognostic factors, such as ulceration and mitotic index, were found to be associated with survival only in the univariate analysis. This may be related to the relatively small number of patients in the present study, compared with the more than 17,000 cases that validate the UICC/AJCC staging system [8]. Other factors related to prognosis only in the univariate analysis that were not confirmed as prognosticators included age, acral lentiginous subtype, Clark level, and microscopic satellitosis.

## Conclusions

This study shows that Brazilian melanoma patients experienced a lower survival rate than the current worldwide average. Our findings confirm the strong association between TNM stage and local recurrence to prognosis. The high prevalence of advanced cases reinforces the importance of local strategies to diagnose melanomas in the early stages, and to treat it definitively. This retrospective study from a single institution has several limitations, such as incomplete data and selection bias and it remains important to perform multi-institutional prospective studies to attain a better understanding of possible socioeconomic and racial disparities in Brazil and Latin America that may affect access to treatment.

## Methods

The study population included all patients diagnosed with melanoma between January 1997 and December 2011 at the Barretos Cancer Hospital (BCH) in Barretos, Sao Paulo State, Brazil. The BCH is a non-profit institution focused on public health and is a high-volume tertiary cancer centre, treating patients from all over the country free of charge [27]. All data were collected from medical records following the appropriate ethical guidelines, and the local ethics committee approved the study (Barretos Cancer Hospital Ethics Committee, #548/2011).

Data analyses were performed with software (SPSS for Windows®, SPSS Inc., Chicago IL, USA). Patients who were previously treated at other institutions were included as long as their records included sufficient information and at least 6 months of follow-up at the BCH. Patients seen for second opinions with incomplete treatment were excluded, as well as all in situ lesions. For survival analysis, at least 24 weeks of follow-up were required. The variables were categorized as demographics (gender, patient origin, age, and skin colour), clinical/histological data (anatomic location of primary tumour; level of serum LDH at first consultation; histological subtype; Breslow depth; Clark level; presence of ulceration; mitotic index; perineural, vascular, and lymphatic infiltration; and peri- and intratumoural lymphocyte infiltration), and treatment-related factors (surgery of primary tumour, sentinel node biopsy, systemic therapy, and radiation therapy).

Indications for sentinel node biopsy were tumour thickness ≥ 0.75 mm. Indications for SNB in tumours between 0.75 mm and 1.00 mm thickness were the presence of mitosis or ulceration. Stage IV and clinically diagnosed stage III were contraindications to SNB.

Skin colour was recorded by the clinician. When this information was missing, it was collected from patient identification, which is registered based on self-report. Clinical stage was determined using the 2009 UICC TNM system [10]. Stages were not stratified into subdivisions for analysis. DSS analyses were performed according to the Kaplan–Meier method, using the log-rank test to compare survival curves. For the continuous variable age, we used the Cox proportional hazards regression method; the variables associated with prognosis (p < 0.05 in univariate analysis) were included in the Cox regression multivariate model analysis if at least 400 cases were available for testing, with the calculation of the hazard ratio (HR) for death and modelling according to variables of interest. Age, TNM stage, and ulceration were used as adjusted model variables, regardless of statistical significance [28]. Statistical significance was set as 5% for all tests.

## Abbreviations

AJCC: American Joint Committee on Cancer
BCH: Barretos Cancer Hospital
DSS: Disease-specific survival
HR: Hazard ratio
LDH: Lactate dehydrogenase
SD: Standard deviation
SNB: Sentinel node biopsy
UICC: International Union Against Cancer
UV: Ultraviolet
